# Hepatitis B Virus Vaccination Coverage in Medical, Nursing, and Paramedical Students: A Cross-Sectional, Multi-Centered Study in Greece

**DOI:** 10.3390/ijerph13030323

**Published:** 2016-03-15

**Authors:** Dimitrios Papagiannis, Zoi Tsimtsiou, Ioanna Chatzichristodoulou, Maria Adamopoulou, Ilias Kallistratos, Spyros Pournaras, Malamatenia Arvanitidou, George Rachiotis

**Affiliations:** 1 Department of Hygiene and Epidemiology, Faculty of Medicine, University of Thessaly, Larissa 41222, Greece; dpapajon@yahoo.gr; 2 Laboratory of Hygiene and Epidemiology, Medical School, Aristotle University of Thessaloniki, Thessaloniki 54124, Greece; zoitsimtsiou@yahoo.gr (Z.T.); vayona@med.auth.gr (M.A.); 3 Department of Microbiology, Medical School, National and Kapodistrian University of Athens, Athens 11527, Greece; jchatzichri@yahoo.gr (I.C.); spournaras@med.uoa.gr (S.P.); 4 Department of Medical Laboratories, Molecular Virology Laboratory, Technological Institution of Athens, Athens 12243, Greece; maryadam@teiath.gr; 5 School of Health Professions, Alexander Technological Educational Institute of Thessaloniki, Thessaloniki 57400, Greece; elikall@phys.teithe.gr

**Keywords:** hepatitis B, vaccination, healthcare, students

## Abstract

Students of health professions are at high risk of hepatitis B Virus (HBV) infection during their clinical training. The aim of this cross-sectional, multi-centered study was to investigate the HBV vaccination coverage in Greek medical, nursing, and paramedical students, to look into their attitudes towards the importance of vaccines and to reveal reasons associated with not being vaccinated. A self-completed, anonymous questionnaire was distributed to 2119 students of health professions in Greece, during the academic year 2013–2014. The HBV vaccination coverage of students was high (83%), being higher among medical students (88.1%, *vs.* 81.4% among nursing and 80.1% among paramedical students; *p* < 0.001). The vast majority of them (95%) have been vaccinated during childhood. In addition, 30% of the unvaccinated students declared fear over HBV safety. Our results indicate that the healthcare students achieved higher reported immunization rates compared to the currently serving healthcare workers, but also to the students of the last decade. The fact that nursing and paramedical students have lower coverage figures underlines the importance of targeted interventions for the different subgroups of healthcare students in terms of educational programs and screening for HBV markers in order to increase HBV vaccination uptake.

## 1. Introduction

Hepatitis B is a well-known global public health problem. There is evidence that 360 million people are chronically infected, of whom almost one million people die annually of hepatitis B virus (HBV)-related liver disease. Recognizing that healthcare workers are in high occupational risk of HBV infection due to their potential contact with blood or body fluids, and possible needle stick injuries, European countries have adopted recommendations for their protection [1]. Since students of health professions (medical, nursing, and paramedical) are the future healthcare workers that are early exposed to the risk of HBV infection during their clinical training, it is recommended that they should also be immunized against HBV (Recommended Vaccines for Health Care Workers [2]).

However, healthcare students’ vaccination coverage against HBV has received limited research attention internationally, especially for the paramedical students. The National Immunization Program in Greece recommends HBV vaccination in healthcare professionals and students [3], although it is not mandatory, as in infants from 1998. Recognizing the importance of the knowledge of the vaccination coverage in the high risk group of healthcare students, only one study has been previously contacted in a single nursing school [4]. However, no statistical data on students’ HBV vaccine coverage are available at a national level in Greece. The aim of this cross-sectional study was to investigate the HBV vaccination coverage in the students of the six largest schools of health professions in Greece. Furthermore, this study aimed to look into the attitudes of healthcare students towards the importance of vaccines and to reveal reasons associated with not being vaccinated.

## 2. Materials and Methods

### 2.1. Participants

All the third- and fourth-year students (*n* = 2119) of the Schools of Medicine, Nursing, and the paramedical health professions of the six largest institutions in Greece (University of Athens, University of Thessaloniki, University of Thessaly, Technological Educational Institute of Athens, Technological Educational Institute of Thessaloniki and Technological Educational Institute of Thessaly) were invited to participate in the study. The study was conducted during the academic year 2013–2014. 

#### Ethics Approval

The protocol of the study was approved by the Steering Committee of the Postgraduate Program: Applied Public Health and Environmental Hygiene of Medical Faculty University of Thessaly (second Assembly 2013; Project Identification Code 01022013).

### 2.2. Questionnaire

A self-administered, anonymous questionnaire (see Appendix) was distributed to participants indicating that its completion was voluntarily. The questionnaire included demographics (sex, age, ethnicity, school of health sciences training) and two questions on student’s attitudes towards the importance of vaccination in terms of public health protection (5-point Liker scale: fully agree, agree, uncertain, disagree, completely disagree). Additionally, the participants were asked whether they had been vaccinated against HBV vaccine (dichotomous answer: yes/no); if they responded positively, they were asked to specify the date of the vaccination and number of HBV vaccine doses they had received. In the case of declaration of no HBV vaccination, the participants were asked to state the reason, answering to a multiple choice question with the following possible answers: lack of time, inertia, use of alternative drugs, perception of not being at risk of serious illness, fear of vaccine side effects, and other. The students were asked to rate the level of information about the safety of the HBV vaccine, choosing among: no information, insufficient information, sufficient, and very good information (dichotomous variable: sufficient/very good *versus* no information/insufficient information). 

### 2.3. Statistical Analysis 

Data were tabulated using the SPSS 21.0 statistical package (SPSS Inc., Chicago, IL, USA). Questionnaire items that were not fully or clearly completed were reported as missing values. Variables were presented as counts and proportions. A chi-square test was used for the univariate analysis of the association between questionnaire items and HBV vaccination. Relative risks (RR) (and their 95% confidence intervals (CI)) were used as the measure of association in univariate analysis. Odds ratio (OR) is the measure of association in multivariate analysis. Logistic regression analysis was used to identify possible factors independently associated with the likelihood of HBV vaccination. *p*-values were considered statistically significant if *p* < 0.05.

## 3. Results

The demographic characteristics of the 1717 students who participated in this study (RR: 81%) are presented in Table 1. The vast majority of the participants (83.0%) were females and 17% were males. Their mean age was 20.9 (SD = 3.00). Regarding School of Health Science training, 42% were nursing students, 30% paramedical, and 28% medical students. Eighty-three percent of the respondents reported that they had been vaccinated with two or three doses of hepatitis B vaccine. The vast majority of them (95%; *n* = 1334) reported that they have been vaccinated during their childhood. The major reason of no vaccination was inertia (60%) or fear (30%) over HBV vaccine safety. 1641 students (96%) agreed that vaccinations are an important tool for the protection of public health, while 1529 students (89%) reported agreement with vaccinations in general.

Univariate analysis (Table 2) showed that students who reported that vaccinations are an important tool to protect public health were more likely to report being vaccinated against HBV compared to their colleagues that disagreed with the importance of vaccinations for the protection of public health (RR 1.30, 95% CI: 1.09–1.55). Analysis of HBV vaccination coverage by school of health sciences training indicated that medical students showed higher HBV vaccination coverage (88.1%) compared to nursing students (81.4%) and paramedical students (80.1%) (Table 2).

When the categorical variable School of Health Sciences Training was transformed to a dichotomous one (medical students *vs.* other), the analysis indicated that students of medical school recorded an increased likelihood of reported HBV vaccination compared to their colleagues from nursing and paramedical departments (RR = 1.09, 95% CI: 1.04–1.13). The students who agree with vaccinations in general recorded an increased likelihood (16%) of an HBV vaccination report than students who disagreed (RR = 1.16; 95% CI: 1.06–1.28). Finally, the age and the sex were not significantly associated with a report of HBV vaccination among students of Health Sciences Schools (Table 2).

Multivariate analysis (Table 3) confirmed that medical students were more likely to report vaccination against HBV compared to their colleagues from nursing and paramedical training (OR = 1.71; 95% CI: 1.25–2.34). Additionally, students who reported that vaccinations are in important mean for the protection of public health were more likely to report HBV vaccination compared to those who disagreed (OR = 2.03; 95% CI: 1.10–3.72).

Finally, students of health sciences who reported a positive opinion towards vaccination in general were more likely to report vaccination against HBV (OR = 1.53, 95% CI: 1.00–2.35).

## 4. Discussion

This is the first, cross-sectional, multi-centered study in Greece investigating HBV vaccination coverage, attitudes of healthcare students towards the importance of vaccines in public health, and reasons associated with not being vaccinated. The coverage of students was high, overcoming the four fifths of the sample. Positive attitudes towards vaccination among healthcare students statistically increased the likelihood of vaccination report significantly. On the other hand, the main reason for not being vaccinated was inertia, while the one third of the unvaccinated students declared fear over HBV safety.

The high HBV vaccination coverage revealed a very promising message, indicating a change in attitudes and practices over the last several years. The future healthcare professionals reported higher vaccination coverage compared with personnel currently working in Greek healthcare settings [5,6,7,8]. In two recent studies, in hospital personnel in Southwestern Greece and in healthcare workers in pediatric departments, the two thirds of the participants were vaccinated (70.9% and 69.2% accordingly) [5,6,7,8], while, in two other studies on healthcare workers in Primary Health Care Centers and in tertiary care hospitals, only half of the personnel were covered towards HBV (55.7% and 56.5% accordingly) [6,7]. Similar findings have been presented in a study of nurses in 17 Greek hospitals where 63.2% were vaccinated against HBV [9]. Interestingly, in an older American study, vaccine acceptance rates were also reported significantly higher among the students than among either the residents or the staff physicians, while the students had attributed their high acceptance rate to compliance to the recommendations of authority figures [10]. Moreover, the higher coverage in students could also indicate a change in attitudes towards the importance of occupational vaccines. Finally, this significant raise in HBV vaccine coverage may also reflect the positive effects in attitudes towards HBV vaccination as a result of its implementation in the mandatory Greek vaccination program in childhood since 1998. Indeed, our study revealed that the vast majority of the vaccinated students have been vaccinated during childhood. A recent study among Italian medical students found a similar pattern of vaccination during childhood. A recent study in Greek adolescents aged 11 to 19 has shown a vaccination coverage (HBV) of 96% [11].

The vaccination coverage figures in our study are comparable with the published results in recent European studies in healthcare students. The findings are also in line with the ones reported in a German study (86.5%) [12], an Italian study of medical students (83.7% tested positive for antibody to hepatitis B surface antigen) [13], two previous Greek studies of non-medical healthcare students from a single institution (82.7%) [14], and in medical and nursing students from a single institution (antibodies to hepatitis B surface antigen were detected for 84.4%) [15]. Moreover, similar results were found in a Turkish study on nursing students (85.3%) [16]. The rates of the students that have received three doses of HBV vaccination reported in the above European studies are higher than those reported within the last five years in other studies worldwide in healthcare students, and specifically in Asia (70.6% in medical students in Pakistan [17], 72.4% medical, dental, and nursing students in Nepal [18], and 21% in students of Medicine, Pharmacy, Dentistry, Nursing Sciences and Medical Technology in Laos [19]), in the Middle East (43.8% in Syrian medical students [20]), and in Africa (18% in medical students in Cameroon [21]). Interestingly, the current vaccination coverage is also higher than the only other previously reported in Greek healthcare (nursing) students a few years ago (65.7%) [4]. Since healthcare students represent a particularly at-risk group for HBV transmission and should be vaccinated as early as possible after the start of their career, these findings illustrate that aiming at increasing hepatitis B vaccination coverage among future healthcare workers is feasible, but still remains a challenge.

Future physicians were found to have statistically significantly higher HBV vaccination coverage compared to nursing and the other paramedical students. Similar findings have been previously reported in physicians compared to nurses and paramedics [22], probably reflecting their higher knowledge level associated with the benefits of vaccination and their more positive attitudes towards vaccination in general. Furthermore, previous research has also shown higher rates of acceptance of mandatory vaccination in Greek physicians compared to other categories of healthcare professionals [6,7].

Looking into the reasons why healthcare students did not report HBV vaccination, inertia was the predominant one, while fear of the vaccines safety was also stated by one third of the uncovered students. Since severe adverse effects following HBV immunization are extremely rare, and severe allergic reactions are expected to occur about once in 1.1 million doses [23], the reported level of fear seems unjustified, possibly implying a knowledge gap on the safety of the HBV vaccine. Therefore, targeted educational programs [24] tailored to the needs of students of health professions are expected to fill possible knowledge gaps about the benefits and risks of HBV vaccination, focusing on the very low probability of serious adverse events, in order to clarify possible misconceptions, develop more positive attitudes towards vaccination, and therefore minimize the reported fear. Moreover, in the effort to increase vaccination coverage and confront inertia, combined strategies that are routinely evaluated could be adopted, taking into consideration the feasibility of mandatory vaccination policies and routine screening for HBV markers in order to further increase vaccination coverage among future healthcare workers. It is noteworthy that a European survey of hepatitis B vaccination policies for healthcare workers reported a diversity of approaches towards the mandatory or recommended policy on HBV vaccination of Students of Health Professions [25].

Some limitations may affect the interpretation of our finding. Our results are based on self-report answers and not on presentation of an official health booklet, including immunization history or the detection of antibodies to hepatitis B surface antigen. However, the validity of results could reinforce the findings of a previous study in medical and nursing students in a tertiary-care hospital in Greece that has shown similar percentages in immunity to hepatitis B (84.4%) after the detection of serum antibodies [16]. In addition, a Greek cross-sectional study among adolescents aged 11–19 years reported that the vaccination coverage for HBV was 96% [26]. Furthermore, using self-reported questionnaires is a method used in numerous previous related studies [6,7,8,14]; for this reason, a comparison of results could be achieved. An additional limitation is that we were not able to obtain information from non-responders. Lastly, the high female/male ratio reflects the gender distribution of the students of Health Professions population in Greece.

## 5. Conclusions

This is the first large scale, multi-centered, cross-sectional study in healthcare students in Greece and one of the few internationally that investigated HBV vaccination coverage in not only medical and nursing students, but also paramedical students. The coverage figures that were found indicate that the healthcare students have achieved higher immunization rates compared to the currently serving healthcare workers and to the students of the last decade. However, it should be noted that a considerable part (17%) of students of Health Professions were unvaccinated against HBV. The fact that nursing and paramedical students have lower coverage figures underlines the importance of targeted interventions for the different subgroups of healthcare students, in terms of educational programs and screening for HBV markers, in order to increase HBV vaccination uptake. Since inertia and fear of the vaccines safety were among the predominant reasons for not being vaccinated, combined strategies should be applied and regularly evaluated in order to help develop positive attitudes towards the value of immunization, increase awareness of the HBV vaccine’s safety, and further increase vaccination coverage among future healthcare workers. In this context the Hospital Infectious Control Committees and the Occupational Health Departments should identify and immunize susceptible students of health professions against HBV. 

## Figures and Tables

**Table 1:** Characteristics of healthcare workers and attitudes towards HBV vaccination.

Characteristics		N/Total (%)
Sex	Male	290/1716 (17%)
	Female	1426/1716 (83%)
Health Science Training	Medical	482/1717 (28%)
	Nursing	716/1717 (42%)
	Paramedical	519/1717 (30%)
Vaccinations are important for the protection of public health and, in particular, of healthcare workers:	I agree	1641/1712 (96%)
	I disagree	71/1712 (4%)
My opinion on vaccination is:	I agree	1529/1713 (89%)
	I disagree	184/1713 (11%)
Have you been vaccinated with HBV vaccine?	Yes	1404/1695 (83%)
	No	291/1695 (17%)
If not, please specify the reason	Inertia	174/291 (60%)
	Fear of vaccine safety	87/261 (30%)
	Lack of time	6/291 (2%)
	I am not at risk of serious illness	24/291 (8%)

**Table 2:** Univariate analysis of vaccination with HBV vaccine.

Variable		N/Total (%)	RR (95% CI)	*p*-Value
Vaccinations are important for the protection of public health	Agree	1354/1620 (83.6%)	1.30 (1.09-1.55)	<0.001
	Disagree	45/70 (64.3%)		
School of Health Science Training (categorical)	Medical	422/479 (88.1%)	--	0.001
	Nursing	572/703 (81.4%)		
	Paramedical	410/512 (80.1%)		
School of Health Science Training (dichotomous)	Medical	422/479 ( 88.1%)	1.09 (1.04–1.13)	<0.001
	Nursing/Paramedical	982/1215 (80.8%)		
Opinion on vaccination in general	Agree	1269/1509 (84.1%)	1.16 (1.06–1.28)	<0.001
	Disagree	131/182 (72.0%)		
Age	<21	818/1002 (81.6%)	0.96 (0.92–1.00)	0.104
	≥21	580/685 (84.7%)		
Sex	Male	231/286 (80.8%)	0.96 (0.91–1.03)	0.300
	Female	1172/1407 (83.3%)		

**Table 3:** Multivariate analysis of HBV vaccination.

Variable		Vaccination with HBV Vaccine (Yes/No)	
		OR (95% CI)	*p*-Value
Vaccinations are important for the protection of public health and, in particular, of healthcare workers	Agree	2.03 (1.10–3.72)	0.021
	Disagree		
Health Science Training	Medical	1.71 (1.25–2.34)	<0.001
	Nursing/Paramedical		
Opinion on vaccination in general	Agree	1.53 (1.00–2.35)	0.047
	Disagree		

## Abbreviations

The following abbreviations are used in this manuscript:

HBV: hepatitis B Virus
CI: confidence interval
RR: response rate
OR: odds ratio

## Appendix

Questionnaire on attitudes and practices related to vaccination against hepatitis B Virus (HBV) among students of Health Professions.

## A. Demographics

**A1**. Age (years) ____________

**A2**. Sex: Male ☐ Female ☐ Nationality ____________

**A3**. School of Health Science Training: Medical ☐ Nursing ☐ Medical laboratories ☐

## B. General attitudes towards vaccinations

**B1.** Vaccinations are an important tool for the protection of public health.

Fully agree ☐
Agree ☐
Uncertain ☐
Disagree ☐
Completely disagree ☐

**B2.** My general opinion about vaccinations is:

Fully agree ☐
Agree ☐
Uncertain ☐
Disagree ☐
Completely disagree ☐

## C. Attitudes and practices about HBV vaccination

**C1**. Have you been vaccinated against hepatitis B Virus?

Yes ☐ No ☐

If yes, please report the number of doses ____________
If no, please specify why
1. Lack of time ☐
2. Inertia ☐
3. Use of homeopathic medicines ☐
4. Not at risk ☐
5. Fear for side effects ☐
Time of vaccination
If yes, please provide the date of HBV vaccination ____________

**C2.** Please rate your information on the safety of HBV vaccines

1. I have no information ☐
2. Insufficient ☐
3. Sufficient ☐
4. Excellent ☐
